# Exosome-Derived Long Non-Coding RNAs as Non-Invasive Biomarkers of Bladder Cancer

**DOI:** 10.3389/fonc.2021.719863

**Published:** 2021-08-19

**Authors:** Quanxin Su, Hao Wu, Ziyi Zhang, Chao Lu, Lifeng Zhang, Li Zuo

**Affiliations:** ^1^Department of Urology, The Affiliated Changzhou No. 2 People’s Hospital of Nanjing Medical University, Changzhou, China; ^2^Department of Graduate School, Dalian Medical University, Dalian, China

**Keywords:** exosomes, lncRNAs, bladder cancer, noninvasive tests, pooled analysis

## Abstract

**Objective:**

As a result of the inconsistency between reports, a meta-analysis was designed to appraise the clinical implications of long non-coding RNAs (lncRNAs) in exosomes for the diagnosis of bladder cancer.

**Methods:**

The PubMed, EMBASE, and Cochrane library databases were searched to identify the relevant literature on lncRNAs in exosomes for bladder cancer diagnosis from database inception to May 2021. The literature was screened according to the inclusion and exclusion criteria, and the Quality Assessment of Diagnostic Accuracy Studies-2 entry tool was applied to evaluate the quality of the literature, and the sources of heterogeneity were explored using meta-regression and subgroup analysis. Stata 14.0 and RevMan 5.3 software were used for statistical analysis.

**Results:**

A total of 23 studies described in 10 articles were included, with a total of 1883 patients with bladder cancer and 1721 patients in the non-cancerous control group. The exosome-derived lncRNAs performed better in the diagnosis of bladder cancer with a pooled sensitivity of 0.74 (95% CI, 0.69-0.77), specificity of 0.76 (95% CI, 0.72-0.80), and area under the curve of 0.83. The heterogeneity between studies was partly as a result of differences in specimen type, number of lncRNAs, lncRNA expression form, and reference gene type. Subgroup analysis showed that the detection efficacy based on the combination of multiple lncRNAs (0.86, 95% CI, 0.82-0.88) was higher than that based on a single lncRNA (0.81, 95% CI, 0.78-0.85), and exosomal lncRNAs with blood as the detection sample had a high diagnostic efficacy (0.86, 95% CI, 0.82-0.86).

**Conclusions:**

Exosome-derived lncRNAs hold great promise as non-invasive diagnostic biomarkers of bladder cancer. However, their clinical value needs to be examined in further comprehensive prospective studies.

## Introduction

Bladder cancer is a common malignancy in urology worldwide and ranks 11th among the most common malignancies throughout the body (1). Approximately 80% of patients with bladder cancer have non-muscle invasive bladder cancer at initial onset with a better prognosis. But they are at risk of recurrence after transurethral resection of the bladder tumor. The remaining patients have muscle-invasive bladder cancer, which is prone to distant metastasis and has a high risk of death (2). A good prognosis for bladder cancer relies on early detection. Cystoscopy and tissue biopsy are the gold standard for the diagnosis of bladder cancer. However, the invasive nature of the procedure and high cost limit its application. Non-invasive and low-cost urine exfoliation cytology is not indicated for low-grade bladder cancer (3). Other currently known bladder cancer tumor biomarkers lack diagnostic sensitivity and specificity, despite their high detection rates. Therefore, it is important to find non-invasive humoral tumor markers with high sensitivity and specificity to establish a safe and effective detection method for the early diagnosis of bladder cancer.

Exosomes are found in many types of body fluids with high abundance. They contain multiple components such as proteins and RNAs and can participate in material and information exchange between cells. Many studies have suggested that exosomes can be used as a new approach for the screening and early diagnosis of tumors. Long non-coding RNAs (lncRNAs) are longer than 200 nucleotides encoding no protein, which play key roles in gene expression, differentiation. In recent years, increasing studies have highlighted the role of lncRNAs in the carcinogenesis of bladder cancer and suggested that lncRNAs might be used as biomarkers in cancer (4, 5). An increasing number of studies have suggested that lncRNAs in exosomes may serve as potential markers for bladder cancer diagnosis. However, we found that several recent studies had discrepancies in their diagnostic accuracy when assessing the diagnostic value of lncRNAs in exosomes in bladder cancer. Chen et al. had a diagnostic specificity of 85% for bladder cancer, while Maryam et al. had a diagnostic sensitivity of 46.67% for bladder cancer (6, 7). Therefore, we performed a meta-analysis to evaluate the comprehensive diagnostic efficacy of lncRNAs in exosomes for patients with bladder cancer.

## Materials and Methods

### Search Strategy

The present meta-analysis was performed in accordance with the guidelines for diagnostic meta-analysis (8). The PubMed, EMBASE, and Cochrane library databases were searched to identify published studies related to the diagnosis of bladder cancer using the detection of lncRNA in exosomes from database inception to May 2021. Search terms included urinary bladder neoplasms, RNA, long non-coding, lncRNA, RNA, long non-translated, long non-coding RNA, long non-protein coding RNA, lincRNA, and exosome. A combination of subject headings and free words was used for searching, and supplementary searches of references from retrieved articles were conducted to ensure comprehensiveness.

### Inclusion and Exclusion Criteria

The inclusion criteria were as follows: (1) published clinical diagnostic studies employing detection of lncRNAs in exosomes for the diagnosis of bladder cancer; (2) study subjects were patients with clinically already pathologically confirmed bladder cancer; and (3) studies provided sufficient data in the full text to calculate sensitivity and specificity. For studies with overlapping data, the study with the superior results was included. The exclusion criteria were as follows: (1) valid data (true positives, false positives, false negatives and true negatives) could not be extracted for merging; (2) non-clinical studies; and (3) reviews, case reports, and meeting abstracts.

### Data Extraction

Data extraction was performed individually by two investigators, with disagreements resolved with the assistance of a third researcher. The extracted contents included: (1) basic characteristics of the study, including the first author, publication year, country of the study, sample size, specimen type, tumor type, tumor stage, tumor grade, lncRNA species, detection methods, and internal reference genes; and (2) diagnostic implications including TP, FP, FN, TN, sensitivity, and specificity.

### Quality Assessment

The Quality Assessment of Diagnostic Accuracy Studies-2 (QUADAS-2) was used to evaluate the quality of the included accuracy trials. The QUADAS-2 tool consists of four evaluation domains: selection of cases, diagnostic tests to be evaluated, gold standard tests, study flow, and progress. Each evaluation area had three or four landmark issues, which were used to evaluate the risk of bias in each field. The first three parts were also used to evaluate the applicability of the documents included. A total score for the seven items of more than four points means that the quality of literature research is high (9).

### Statistical Methods

The QUADAS-2 evaluation function in RevMan 5.3 was used to evaluate the quality of the included studies for diagnostic accuracy studies, and the pooled sensitivity, specificity, positive likelihood ratio (PLR), negative likelihood ratio (NLR), and diagnostic odds ratio (DOR) were calculated using Stata 14.0. The size of between-study heterogeneity was estimated using I^2^; a fixed effects model was used if I^2^ < 50%, which was considered to indicate small study heterogeneity, and a random effects model was used if I^2^ > 50%, which was considered to indicate substantial heterogeneity (10). The cumulative receiver operating characteristic curve (SROC) was plotted, and the area under the curve (AUC) was calculated to evaluate the diagnostic experimental value. The post-diagnostic effect after the pooled analysis was assessed using Fagan’s nomogram. Meta-regression, subgroup analysis, and sensitivity analysis were used to analyze the sources of heterogeneity. Publication bias was assessed using Deek’s funnel plot asymmetry test, with P < 0.10 indicating significant bias (11).

## Results

### Search Results and Included Literature

**Figure 1:**
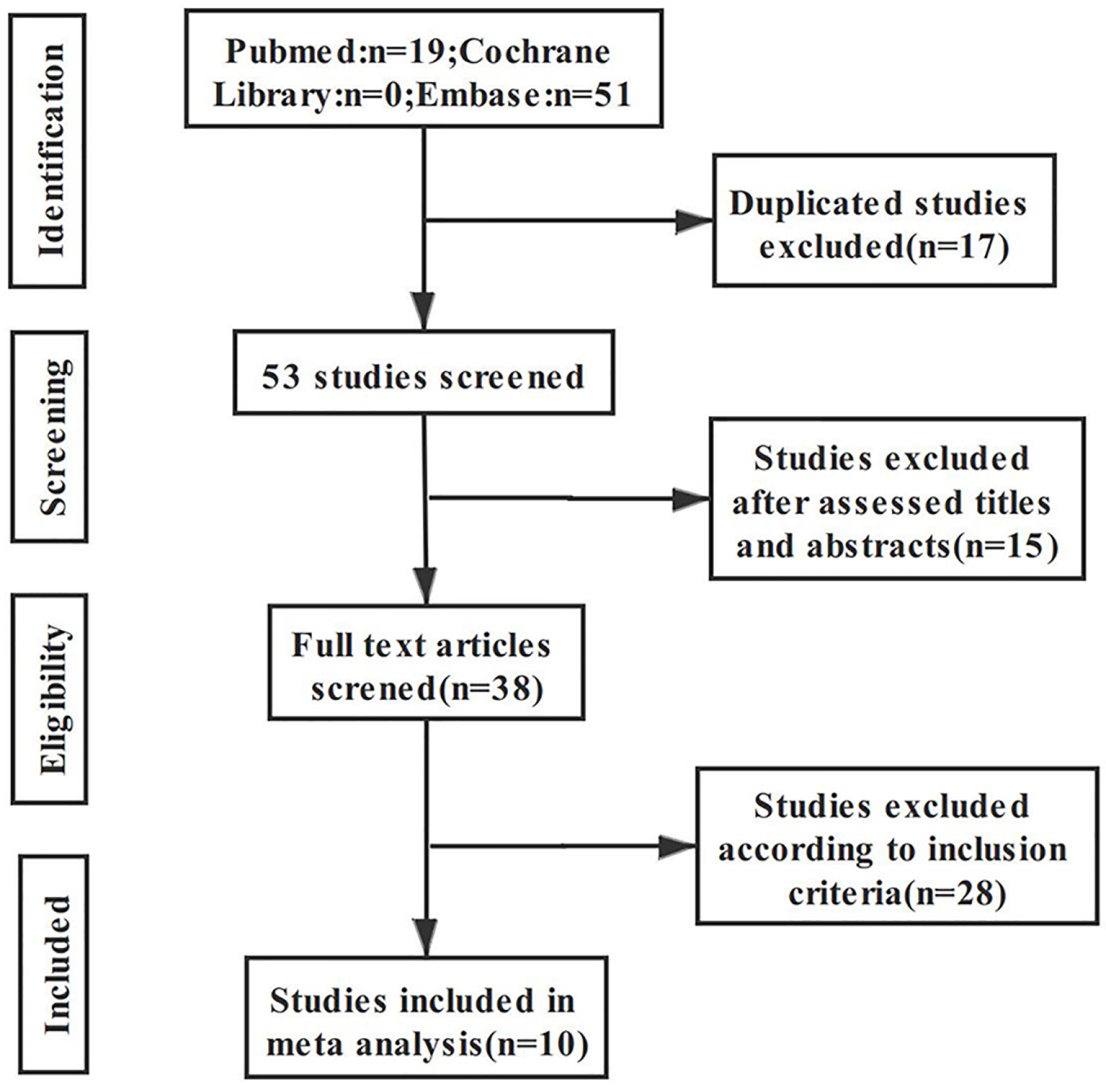
The flow diagram of this meta-analysis.

A total of 70 articles were retrieved through the search strategy; after excluding 17 duplicate articles and 15 articles after primary screening of the title, 38 articles remained for further assessment. The remaining articles were subjected to full-text assessment; 28 articles were further excluded due to ineligibility, resulting in a total of 10 articles being included in the final analysis. The literature screening process and results are shown in **Figure 1**.

### Literature Characteristics and Quality Assessment

Twenty-three studies from 10 articles published between 2017 and 2021 were included in this meta-analysis (6, 7, 12–19). The included population comprised 1883 patients with bladder cancer and 1721 control patients. In terms of sample selection, 18 studies extracted exosomes from urine, and five studies extracted exosomes from blood. Only five studies utilized multiple lncRNAs to detect bladder cancer in combination, and 18 employed a single lncRNA for detection. In terms of patient tumor stage, 20 studies included both patients with NMIBC and patients with MIBC, and all studies involved both low- and high-grade bladder cancer. In terms of lncRNA detection, 22 studies used real-time reverse transcription polymerase chain reaction (qRT-PCR) to detect the expression levels of lncRNAs and convert the lncRNA concentrations to the expression levels of internal reference genes. The basic information of the included studies is presented in **Table 1**.

**Table 1:** Characteristics of studies included in the meta-analysis.

Author	Year	Country	LncRNAs	Sample	Case/Control	Cancer type	Control type	Tumor stage	Tumor grade	Expression in BC	Reference gene	TP	FP	TN	FN
Mei Xue	2017	China	UCA1	Serum	30/30	BC	healthy volunteers	16Ta-T1+14T2-T4	9 Low + 21high	up-regulated	ATCB/GAPDH	24	5	25	6
Rui Zheng	2018	China	PTENP1	Urine	50/60	BC	healthy volunteers	41Ta-T1+9T2-T4	35G1-2 + 15G3-4	down-regulated	GAPDH	37	9	51	19
Yao Zhan	2018	China	MALAT1	Urine	80/80	BC	healthy volunteers	50Ta-T1+30T2-T4	39Low + 41high	up-regulated	GAPDH	63	26	54	17
			PCAT-1							up-regulated		57	16	64	23
			SPRY4-IT1							up-regulated		70	28	52	10
			3 lncRNAs ^a^							NA		50	12	68	30
			MALAT1	Urine	104/104	BC	healthy volunteers	61Ta-T1+43T2-T4	46Low+ 58high	up-regulated	GAPDH	75	16	88	29
			PCAT-1							up-regulated		75	19	85	29
			SPRY4-IT1							up-regulated		69	24	80	35
			3 lncRNAs ^a^							NA		73	15	89	31
Jiansong Wang	2018	China	H19	Serum	52/52	BC	9 BPH + 15 urolithiasis+18 cystitis	29T1-T2+24T3-T4	28Low+24high	up-regulated	GAPDH	39	11	41	13
Fatemeh Yazarlou	2018	Iran	UCA1-201	Urine	59/49	TCC	24 normal + 11bladder stone + 6obstructive uropathy + 8BPH	NA	20Low+28high	down-regulated	5S rRNA	51	22	27	8
			UCA1-203							up-regulated		43	23	26	16
			MALAT1							up-regulated		37	15	34	22
Shujun Zhang	2019	China	3 lncRNAs ^b^	Serum	160/160	BC	healthy volunteers	84Ta-T1+76T2-T4	66Low+94high	NA	GAPDH	128	40	120	32
			3 lncRNAs ^b^	Serum	100/100	BC	healthy volunteers	56Ta-T1+44T2-T4	48Low+52high	NA	GAPDH	14	1	9	16
Maryam Abbastabar	2020	Iran	ANRIL	Urine	30/10	BC	healthy volunteers	20T1 + 10T2	13Low+17high	up-regulated	5s rRNA	13	1	9	17
			PCAT-1							up-regulated		62	14	66	18
Haiming Huang	2021	China	MIR205HG	Urine	80/80	BC	healthy volunteers	64Ta-T1+16T2-T4	35Low+45high	up-regulated	5s rRNA	63	32	48	17
			GAS5							down-regulated		54	10	70	26
			2 lncRNAs							NA		206	50	116	36
Changhao Chen	2021	China	ELNAT1	Serum	242/166	BC	healthy volunteers	79T1+163T2-T4	65Low+177high	up-regulated	NA	23	2	8	7
Mohammad Sarf	2021	Iran	TUG-1	Urine	30/10	BC	healthy volunteers	20Ta-T1+10T2	13Low+17high	up-regulated	5s rRNA				

3 lncRNAs ^a^, MALAT1+PCAT-1+SPRY4-IT1.

3 lncRNAs ^b^, PCAT-1+UBC1+SNHG16.

2 lncRNAs, MIR205HG + GAS5.

TCC, transitional cell carcinoma.

BC, bladder cancer.

“NA” means not available.

**Figure 2:**
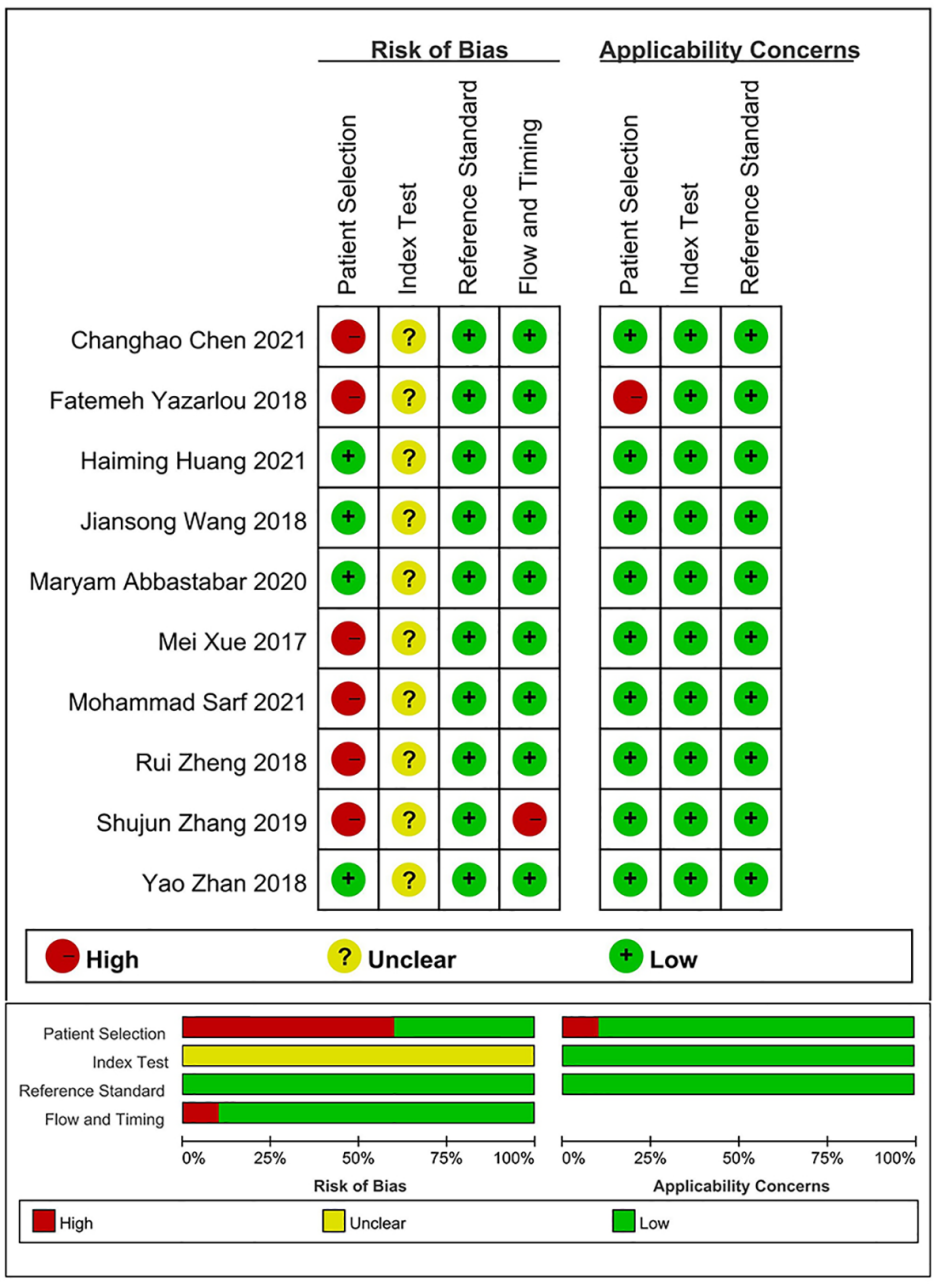
QUADAS-2 entries for evaluation of literature quality.

The results of the evaluation of the quality of articles performed using QUADAS-2 (**Figure 2**) showed that all 23 included studies reached medium to high quality level, but there was some bias in case selection and diagnostic tests to be evaluated. This may be due to the fact that the time of testing was not defined for some of the included samples, and most studies did not explicitly state whether the reviewers interpreted the results of the trials to be evaluated without knowing the results of the gold standard assessment.

### Diagnostic Performance

**Figure 3:**
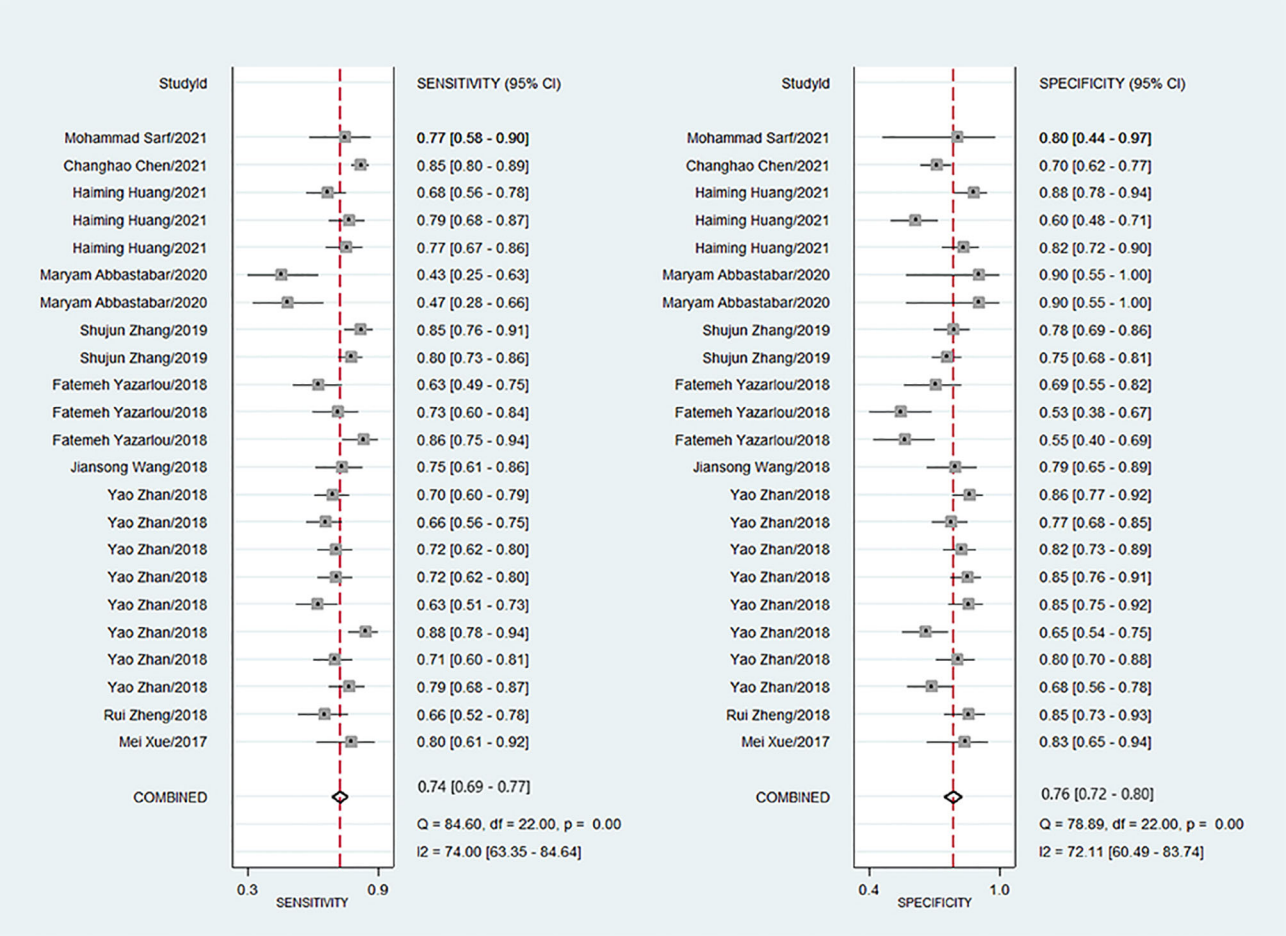
Forest plots of sensitivity and specificity for exosome derived lncRNAs in the diagnosis of bladder cancer.

**Figure 4:**
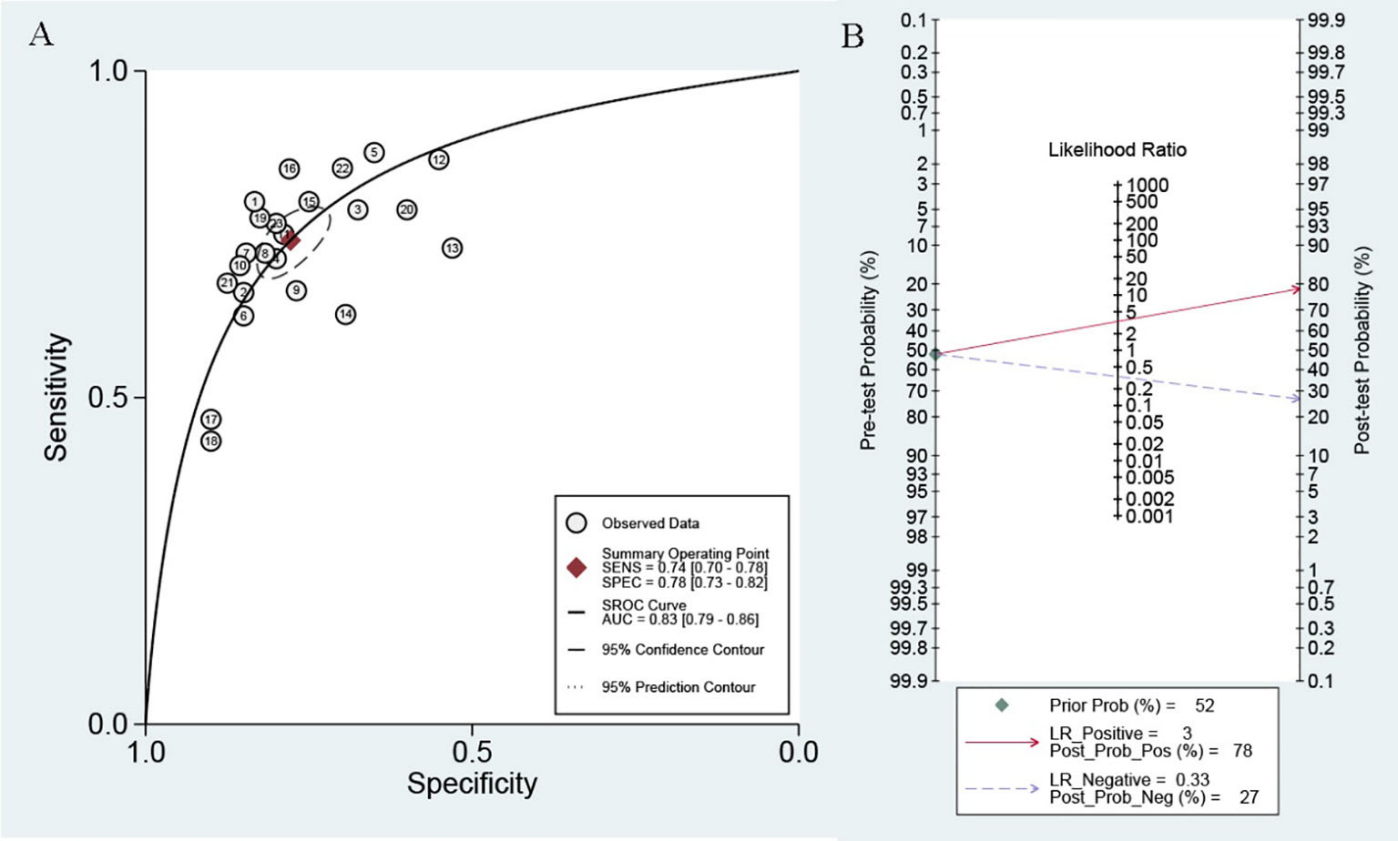
**(A)** SROC curve with pooled estimates of sensitivity, specificity and AUC of overall studies. **(B)** Fagan’s nomogram for evaluation of post-test probabilities based on pooled estimates of PLR and NLR of overall studies.

Because of significant heterogeneity (P < 0.01) among the studies in terms of sensitivity (I^2^ = 74.00%) and specificity (I^2^ = 72.11%), a random effects model was chosen to combine effect sizes. The specific results are shown in **Figure 3**. The pooled results across all 23 studies were: sensitivity, 0.74 (0.69–0.77) and specificity, 0.76 (0.72–0.80). The PLR was 3.3 (2.8–3.9), and the NLR was 0.33 (0.29–0.38), indicating that bladder cancer patients had 3.8 times more positive test results than healthy individuals. The DOR was 10 (8–12), indicating that lncRNAs in exosomes can be used to distinguish bladder cancer patients from controls (**Table 2**). The AUC was 0.83 (0.79–0.86), indicating that lncRNAs in exosomes could be a better diagnostic indicator for bladder cancer, and the results are shown in **Figure 4A**. According to the Fagan plot (**Figure 4B**), the pre-test probability was 52%, the post-test probability of a positive bladder cancer test was 78%, and the post-test probability of a negative bladder cancer test was 27%, which illustrated that the post-test probability and likelihood ratio were moderate.

**Table 2:** Subgroup analysis of lncRNA for the diagnosis of bladder cancer.

Analysis	No. of studies	SEN (95% CI)	SPE (95% CI)	PLR (95% CI)	NLR (95% CI)	DOR (95% CI)	AUC (95% CI)
**Overall**	23	0.74 (0.69–0.77)	0.76 (0.72–0.80)	3.3 (2.8–3.9)	0.33 (0.29–0.38)	10 (8–12)	0.83 (0.79–0.86)
**Sample types**							
Urine-based	18	0.72 (0.67–0.76)	0.78 (0.72–0.83)	3.3 (2.7–4.1)	0.36 (0.32–0.41)	9 (7–11)	0.81 (0.77–0.84)
Blood-based	5	0.82 (0.78–0.86)	0.75 (0.70–0.79)	3.3 (2.8–4.0)	0.24 (0.19–0.29)	14 (10–19)	0.86 (0.82–0.86)
**Reference types**							
GAPDH	12	0.75 (0.70–0.79)	0.79 (0.75–0.83)	3.6 (3.1–4.1)	0.32 (0.28–0.37)	11 (9–14)	0.84 (0.80–0.87)
non GAPDH	11	0.73 (0.64–0.80)	0.76 (0.66–0.83)	3.0 (2.2–4.1)	0.36 (0.28–0.46)	8 (5–13)	0.81 (0.77–0.84)
**lncRNA types**							
Single-lncRNA	18	0.74 (0.69–0.79)	0.76 (0.70–0.81)	3.1 (2.6–3.7)	0.34 (0.29–0.40)	9 (7–11)	0.81 (0.78–0.85)
Multiple-lncRNA	5	0.74 (0.66–0.81)	0.82 (0.77–0.87)	4.2 (3.3–5.3)	0.32 (0.25–0.41)	13 (10–18)	0.86 (0.82–0.88)
**Expression types**							
up-regulated in BC	16	0.72 (0.67–0.77)	0.79 (0.74–0.83)	3.4 (2.8–4.1)	0.35 (0.30–0.41)	10 (7–12)	0.82 (0.79–0.85)
non up-regulated in BC	7	0.78 (0.71–0.84)	0.75 (0.65–0.83)	3.1 (2.3–4.3)	0.29 (0.23–0.36)	11 (8–15)	0.84 (0.80–0.87)

### Meta-Regression and Subgroup Analysis

**Figure 5:**
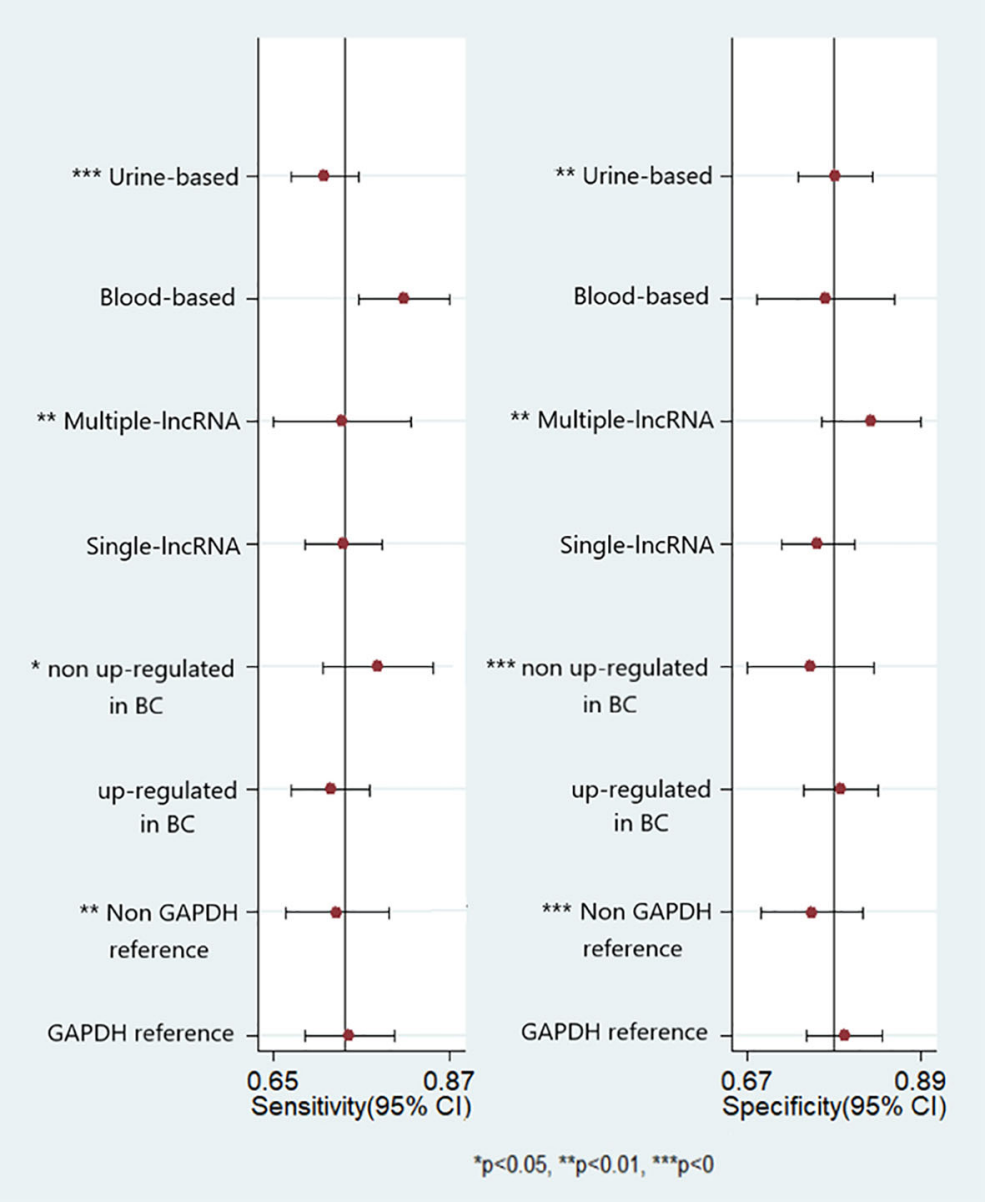
Forest plots of multivariable meta-regression analyses for sensitivity and specificity (vertical lines signify pooled estimates of sensitivity and specificity respectively).

Potential sources of heterogeneity were explored using a meta-regression analysis. As shown in **Figure 5**, specimen type, number of lncRNAs, lncRNA expression form, and reference gene type were the main sources of heterogeneity in exosomal lncRNA detection in bladder cancer. Subgroup analysis was further performed according to the above factors, and the pooled results of diagnostic value in different subgroups are shown in **Table 2**. Among them, exosomal lncRNAs in blood samples showed higher sensitivity, DOR, and AUC (sensitivity = 0.82, DOR = 14, AUC = 0.86). The combination of multiple lncRNAs had better predictive ability than a single lncRNA (DOR = 13, AUC = 0.86).

### Sensitivity Analysis and Publication Bias

**Figure 6:**
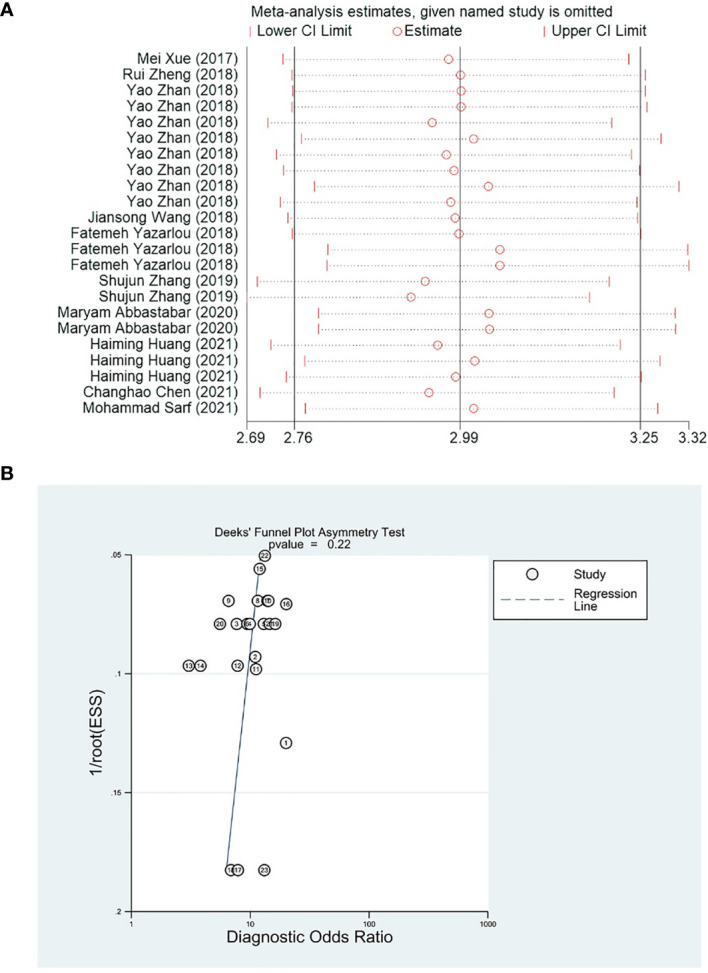
**(A)** Forest plots of sensitivity analysis. **(B)** Deeks’ funnel plot asymmetry test.

Removing single studies one by one, the difference between the pooled effect size of the remaining studies and the total effect size was observed for sensitivity analysis. The results showed that the outcome measures did not change significantly after removal, which indicated that the present meta-analysis was robust (**Figure 6A**). Deek’s funnel plot was used to assess the potential publication bias of the included studies, and suggested that there was no publication bias in the included studies (p = 0.22) (**Figure 6B**).

## Discussion

Bladder cancer is one of the most common malignancies of the urinary system. Recently, with increased understanding of the molecular mechanisms of tumorigenesis, many studies of bladder cancer diagnosis, recurrence, and progression prediction have been conducted, mainly focusing on the abnormalities at the gene, protein, and molecular levels. Several studies have shown that nuclear matrix protein 22, telomerase, vascular endothelial growth factor, hyaluronidase, and proliferation-associated nuclear antigen Ki‐67 have some value in the diagnosis and prognosis of bladder cancer. However, all of the above markers lack sufficient sensitivity or specificity to change the current situation regarding bladder cancer diagnosis based on cystoscopy (20–22). Unfortunately, cystoscopy is an invasive examination with high cost and low patient acceptance, which makes it difficult to be widely used in clinical practice. Therefore, searching for non-invasive bladder cancer tumor markers with high sensitivity and high specificity has become the focus of current clinical attention.

Liquid biopsy is non-invasive, cost-effective, and convenient compared with traditional bladder cancer detection methods, and its sensitivity for tumor diagnosis is higher (23, 24). Exosomes are spherical or ellipsoid shaped secretory vesicles containing bioactive substances, such as nucleic acids and proteins, which are actively produced and secreted by tissue cells. There are some protein molecules on their surface that can act as ligands to specifically bind to the receptor molecules on the surface of target cells. After exosomes fuse with the target cell membrane, the bioactive substances they carry are transferred to the target cell to activate or inhibit the related signaling pathways within the target cell, ultimately affecting the biological function of the target cell (25, 26). Since Trams et al. first found that a subset of tumor cell lines and normal cell lines are able to release exosomes into culture medium, tumor-derived circulating exosomes have attracted increasing attention as a promising alternative to liquid biopsy in non-invasive cancer diagnosis and monitoring treatment response (27).

LncRNAs are non-coding RNAs longer than 200 nucleotides and have limited potential to encode proteins. Relevant studies have reported that lncRNAs account for 3.36% of the total RNA content of exosomes (28). LncRNAs derived from exosomes can mediate intercellular communication signaling molecules to regulate tumor cell proliferation, invasion, and migration, and promote cancer development. Many studies have found that lncRNAs in exosomes play an important role in the early diagnosis of tumors. Zhang et al. found that exosomal Mala T-1 expression was significantly higher in patients with NSCLC than in healthy controls, and the increased exosomal Mala T-1 expression significantly correlated with TNM stage and lymphatic metastasis (29). A study by Mustafa et al. found that lncRNA p21 in the serum exosomes could be used to discriminate between patients with prostate cancer and prostatitis (30). Therefore, an increasing number of researchers believe that lncRNAs of exosomal origin are promising novel diagnostic biomarkers for bladder cancer. We performed a meta-analysis to explore the diagnostic performance of exosome-derived lncRNAs as non-invasive diagnostic biomarkers for bladder cancer.

To the best of our knowledge, this is one of the few evidence-based meta-analyses focused on elucidating the diagnostic significance of exosome-derived lncRNAs in bladder cancer. Our report validated the potential diagnostic performance of exosome-derived lncRNAs as non-invasive biomarkers for discriminating bladder cancer from controls, with a pooled sensitivity of 74% and pooled specificity of 76%. In addition, a DOR value of 10 (8–12) indicated that patients with a positive test for lncRNA of exosomal origin had 10-fold higher odds of developing bladder cancer than controls. Because of the threshold effect between studies, the SROC curve was a better method to evaluate the pooled diagnostic accuracy for discrimination between cases and controls. The AUC of 0.83 suggested high diagnostic accuracy.

Heterogeneity is a potential concern, which affects the interpretation of pooled effects and meta-analysis results. Although we developed strict inclusion and exclusion criteria to identify eligible studies, heterogeneity still existed due to the presence of potential confounding factors. We performed meta-analysis and subgroup analyses to explore potential sources of heterogeneity. Our results showed that factors such as specimen type, number of lncRNAs, lncRNA expression form, and reference gene type all influenced pooled sensitivity and specificity, suggesting that the above factors may be major sources of heterogeneity in lncRNA detection in bladder cancer. The specimen type appeared to contribute to the heterogeneity of circulating lncRNA detection, with a pooled AUC for the diagnostic performance of blood-derived exosomal lncRNA of 0.86. This may be due to the fact that urine contains more deposits, and the heterogeneous cellular components may affect its use as a reliable biomarker. A diagnostic model consisting of multiple lncRNAs appeared to have better diagnostic performance, which may be because the development of bladder cancer was itself the result of a complex multistage process of genomic and epigenetic abnormalities, which should also be affected by multiple lncRNAs. Reference gene type appeared to be another possible source of heterogeneity, with a pooled AUC of 0.84 for GAPDH as the reference gene and 0.81 for a non-GAPDH reference gene. This may be attributed to the different sample situations and test conditions between studies. As reference gene type differences and mechanisms are not well understood, extensive investigations are warranted to further confirm whether such differences are truly present. In terms of lncRNA expression, up-regulated lncRNAs were less predictive than those which non up-regulated. It is worth mentioning that UCA1 has been cloned from human bladder TCC cell line. Xue et al. found that the expression levels of UCA1 in the exosomes of bladder cancer patients were higher. Yazarlou et al. further investigated the expression of two splicing variants of UCA1, UCA1-201 and UCA1-203, in tumor patients. The results showed up-regulation of UCA1-203 while down-regulation of UCA1-201 in TCC samples compared with normal subjects. Such different pattern of expression of these two variants might imply specific roles for them which should be assessed in future studies.

Wang et al. performed an impressive meta-analysis of the diagnostic value of lncRNAs in bladder cancer (31). They reported a pooled sensitivity of 0.76 (0.72 - 0.80) and pooled specificity of 0.77 (0.73 - 0.81) for lncRNAs of exosomal origin in their subgroup analysis. These results support our conclusions. However, in terms of heterogeneity analysis, they did not perform subgroup analysis of reference gene categories and expression forms (up - or downregulated) of lncRNAs in bladder cancer patients. In addition, we found that lncRNAs were stably present in exosomes from serum or urine in each study. The mechanism may be that the membrane structure of exosomes could act as a protective membrane to protect these molecules from degradation. Given the stability of lncRNAs in exosomes and the simplicity and repeatability of detection of serum and urine samples, we deduced that exosome-derived lncRNAs may also be used as clinical biomarkers for other diseases and might be a desirable material for basic research and clinical testing.

The present meta-analysis has several limitations that need to be addressed. First, the significant statistical heterogeneity in our analysis with respect to specimen type, number of lncRNAs, lncRNA expression form, and reference gene type will have an inevitable influence on the results. Second, a good biological marker needs to be able to distinguish cancer from other diseases with similar symptoms. However, most studies included in this meta-analysis simply tried to distinguish bladder cancer patients from healthy populations, without involving bladder cancer patients with similar symptoms. In addition, the populations included in this meta-analysis were mostly Asian with a smaller Caucasian population, with no relevant data on African populations. Finally, although all studies provided information on tumor stage and grade, most did not provide cut-off values; further comprehensive studies are needed to address this issue.

## Conclusion

Our comprehensive analysis confirmed that exosome-derived lncRNAs might serve as potential clinical biomarkers for bladder cancer diagnosis with high AUC values. However, their clinical value still needs to be tested in further comprehensive prospective studies.

## Data Availability Statement

The original contributions presented in the study are included in the article/supplementary material. Further inquiries can be directed to the corresponding authors.

## Author Contributions

All authors contributed to the article and approved the submitted version.

## Funding

This study was supported by grants from Changzhou Sci & Tech program (CJ20190100), the Young Scientists Foundation of Changzhou No. 2 People’s Hospital (YJRC202039; 2019K008), the Innovation team funding (XK201803), the Young Talent Development Plan of Changzhou Health Commission (No. CZQM2020065), the Faculty-level subject funding (YJXK202013), the Top Talent Project (RC201620), and the National Natural Science Foundation (No. 81902565).

## Conflict of Interest

The authors declare that the research was conducted in the absence of any commercial or financial relationships that could be construed as a potential conflict of interest.

## Publisher’s Note

All claims expressed in this article are solely those of the authors and do not necessarily represent those of their affiliated organizations, or those of the publisher, the editors and the reviewers. Any product that may be evaluated in this article, or claim that may be made by its manufacturer, is not guaranteed or endorsed by the publisher.
